# Prediction of Cardiovascular Risk Using Nonalcoholic Fatty Liver Disease Scoring Systems

**DOI:** 10.3390/healthcare9070899

**Published:** 2021-07-15

**Authors:** Ye-Na Kweon, Hae-Jin Ko, A-Sol Kim, Hye-In Choi, Ji-Eun Song, Ji-Yeon Park, Sung-Min Kim, Hee-Eun Hong, Kyung-Jin Min

**Affiliations:** 1 Department of Family Medicine, Kyungpook National University Hospital, Daegu 41944, Korea; yenakwon@naver.com (Y.-N.K.); blbr@naver.com (H.-I.C.); miniev@naver.com (J.-Y.P.); hhe8824@naver.com (H.-E.H.); 2 Department of Family Medicine, School of Medicine, Kyungpook National University, Daegu 41944, Korea; deepai@knu.ac.kr; 3 Department of Family Medicine, Kyungpook National University Chilgok Hospital, Daegu 41404, Korea; love2uje@naver.com (J.-E.S.); kimsungmin83@gmail.com (S.-M.K.); bttbmkj@hanmail.net (K.-J.M.)

**Keywords:** nonalcoholic fatty liver disease, Fatty Liver Index, Hepatic Steatosis Index, Simple NAFLD Score, comprehensive NAFLD Score, Framingham Risk Score

## Abstract

This study aimed to determine whether nonalcoholic fatty liver disease (NAFLD) is an independent risk factor for CVD and to identify the most useful NAFLD diagnostic tool for predicting CVD. Data from a total of 23,376 Korean adults without established CVD were analyzed. Cardiovascular risk was calculated using the Framingham Risk Score (FRS) 2008. The presence of NAFLD was defined as moderate-to-severe fatty liver disease diagnosed by ultrasonography. Scores for fatty liver were calculated using four NAFLD scoring systems (Fatty Liver Index, FLI; Hepatic Steatosis Index, HSI; Simple NAFLD Score, SNS; Comprehensive NAFLD Score, CNS), and were compared and analyzed according to cardiovascular risk group. Using the FRS, 67.4% of participants were considered to be at low risk of CVD, 21.5% at intermediate risk, and 11.1% at high risk. As the risk of CVD increased, both the prevalence of NAFLD and the score from each NAFLD scoring system increased significantly (*p* < 0.001). In the unadjusted analysis, the CNS had the strongest association with high CVD risk; in the adjusted analysis, the FLI score was most strongly associated with high CVD risk. Fatty liver is an important independent risk factor for CVD. Therefore, the available NAFLD scoring systems could be utilized to predict CVD.

## 1. Introduction

Nonalcoholic fatty liver disease (NAFLD) has become one of the most common liver diseases worldwide with a current estimated global prevalence of 30% in adults [1]. NAFLD is associated with obesity, insulin resistance, dyslipidemia, and type 2 diabetes mellitus [2], and is therefore considered the hepatic manifestation of the metabolic syndrome [3]; it is also associated with lifestyle factors including high-fat diets, high calorie intake, and lower levels of physical activity [4]. NAFLD is defined as a fat content of more than 5–10% of liver volume; fat accumulation results from increased fat synthesis and/or delivery, and decreased fat export and/or oxidation. A NAFLD diagnosis can only be made in the absence of a history of significant alcohol consumption and other causes of chronic liver disease [5]. NAFLD encompasses a spectrum of diseases ranging from the first stage of simple steatosis, to steatohepatitis with or without fibrosis and progressing to cirrhosis and hepatocellular carcinoma [6,7]. At the stage of simple hepatic steatosis, NAFLD is reversible if lifestyle modifications, such as dietary interventions, increases in physical activity, or weight loss, are made. Although the natural clinical course of simple hepatic steatosis is benign, some patients may further progress to have an advanced stage of liver disease, and once at the stage of cirrhosis, NAFLD is irreversible: it is therefore important to diagnose and manage NAFLD before it reaches the irreversible stage [8].

NAFLD patients are at risk of cardiovascular disease (CVD); therefore, risk factors for NAFLD are also important risk factors in the development of CVD [4]. Recently, studies have shown a relationship between NAFLD and CVD, with NAFLD patients having an increased risk of CVD development [9,10]. According to a recent meta-analysis of observational and retrospective studies involving 34,043 adults by Claudio et al., compared with patients without NAFLD, patients with NAFLD have a 1.64-fold higher risk of both fatal and non-fatal cardiovascular events, while patients with more severe liver disease have a 2.58-fold higher risk of cardiovascular events [10]. Therefore, it appears that there is a positive correlation between NAFLD stage and CVD risk.

This association may result from processes involved in NAFLD pathogenesis. In NAFLD, oxidative stress and inflammation in the liver induce the progression of NAFLD from simple steatosis toward the more severe form of nonalcoholic steatohepatitis [11]. The inflammatory response is a key mechanism linking NAFLD with CVD: inflammation is a critical factor in the development of atherosclerosis symptoms, including the formation of fatty streaks, formation of atherosclerotic plaques, and rupture of plaques accompanied by blood clots [10]. Since NAFLD is an independent and important risk factor for CVD, it is important to diagnose NAFLD early and establish an effective treatment strategy.

Liver biopsy is the gold standard method for diagnosing NAFLD [12]. However, liver biopsies are not cost-effective and are invasive, posing a risk of side effects such as pain, bleeding, and infection, making it clinically difficult to perform biopsies in all NAFLD patients [13]. Therefore, in clinical settings, various tools can be applied instead of liver biopsy, such as imaging tests, liver ultrasound, liver fibrosis tests, CT or MRI, and scoring systems calculated from biochemistry results [12].

This study aimed to determine whether NAFLD is an independent risk factor for CVD and identify the most useful NAFLD diagnostic tool for predicting CVD.

## 2. Materials and Methods

### 2.1. Ethics Statement

This study was approved by an Institutional Review Board (IRB) at a local hospital, Republic of Korea (protocol no. KNUH 2020-05-061). Anonymous and de-identified data were used for the analysis; therefore, informed consent was not obtained.

### 2.2. Selection of Study Subjects

A retrospective study was performed using data from 28,897 Korean patients who visited Kyungpook National University Hospital Health Promotion Center from January 2011 to August 2017. The inclusion criteria for this study were as follows: (1) subjects aged 30–74 who visited the hospital for health screening during the study period; (2) those who performed all examinations including abdominal ultrasonography and laboratory tests and completed questionnaires on socio-demographics and health-related characteristics; (3) those who had no previous history of any established CVD (e.g., stroke, myocardial infarction, or angina pectoris). Exclusion criteria were as follows: (1) significant consumption of alcohol (defined as more than 210 g/week for males and 140 g/week for females); (2) positive serologic markers for hepatitis B or C viruses, or human immunodeficiency virus; (3) abnormal ultrasonography liver findings (i.e., chronic liver disease, cirrhosis, suspected hepatocellular carcinoma, hepatic mass, or signs of Clonorchis sinensis); (4) presence of thyroid disease, including thyroid cancer, hyperthyroidism, hypothyroidism, or thyroid hormone replacement therapy; (5) absence of questionnaire data or anthropometric measurements; and/or (6) patients with any history of taking drugs (glucocorticoids, tamoxifen, or tetracycline) or supplements that may influence liver enzymes or induce or reduce fatty liver changes. In total, 23,376 patients (13,583 males and 9793 females) were eligible for the analysis.

### 2.3. Anthropometric Measurement

Anthropometric measurements were taken with the participants wearing light clothing with bare feet after at least a 12 h fast. Height was measured by standing on the measuring instrument, matching the shape of the soles of the feet, keeping the knees straight, and looking straight ahead without leaning the back on the stand. A comfortable posture without applying any force to the body was maintained while measuring height and weight. Waist circumference was measured at the middle of the lowermost part of the ribs and the highest part of the pelvis while exhaling comfortably after spreading both feet about 25 to 30 cm to spread weight evenly. Body mass index (BMI) was calculated as the patient’s weight in kilograms divided by the patient’s height in meters squared. Blood pressure was measured after sitting on a chair for 5 min with validated electronic devices.

### 2.4. Demographic Characteristics

Demographic characteristics, including past medical history, were obtained through a health screening questionnaire. Hypertension was recorded if the patient reported diagnosis of hypertension by a physician or treatment with antihypertensive medication, or if a blood pressure of ≥140/90 mmHg was measured. Diabetes was recorded if the patient reported diagnosis of diabetes by a physician or treatment with anti-diabetic drugs, or if a fasting plasma glucose level of ≥126 mg/dL was measured. Dyslipidemia was recorded if the patient reported treatment with cholesterol-lowering medication or if a total cholesterol level of ≥200 mg/dL, a high-density lipoprotein (HDL) cholesterol level of <45 mg/dL in males or <50 mg/dL in females, a low-density lipoprotein (LDL) cholesterol level of ≥130 mg/dL, or a triglyceride level of ≥150 mg/dL was measured [14]. Smoking status was classified as never, ex-, or current smoker according to lifetime exposure to cigarettes. Alcohol consumption was classified as non- or moderate drinker according to the converted quantitated alcohol consumption by types of beverages, frequency of drinking, and average amount of alcohol consumed on each event per week. Nondrinker included lifetime abstainers who drank fewer than 12 drinks in their lifetime or former drinkers who drank at least 12 drinks in any one year in their lifetime but none in past year. Moderate drinker included participants consuming more than at least 12 drinks in the past year, and fewer than 210 g/week for males and 140 g/week for females. Exercise status was classified as regular and non-exerciser according to the duration, frequency, and type of exercise. Regular exercise was defined as engagement in physical activity for at least 30 min twice or more per week.

### 2.5. Laboratory Tests

Laboratory parameters were measured after a minimum of a 12 h fast. Values outside extreme outliers were considered missing values. White blood cells, hemoglobin, erythrocyte sedimentation rates, and thyroid-stimulating hormone levels were measured, including blood sugar, triglycerides, and HDL cholesterol. Serum aspartate transaminase (AST), alanine transaminase (ALT), serum alkaline phosphatase, serum bilirubin, serum total protein, and albumin were measured. In addition, hepatitis B antigen, hepatitis C antibody, and human immunodeficiency virus antibodies were checked to diagnose infectious diseases such as hepatitis, and serum creatinine and glomerular filtration rate were measured for renal function evaluation.

### 2.6. Liver Ultrasonography

In all patients, liver ultrasonography was performed by a skilled radiologist after 12 h of fasting. The severity of fatty liver disease was assessed based on echogenicity, the echoic tissue amplitude of the liver and kidneys, the degree of echo penetration, and the degree to which the vascular structures of the liver were defined. Fatty liver was classified as mild, moderate, or severe based on standard criteria [15,16]. Steatosis was graded as follows: Grade 0, the echotexture of the liver is normal; Grade 1, visualization of the diaphragm and the portal vein wall is normal but there is a slight and diffuse increase of liver echogenicity; Grade 2, there is a moderate increase of liver echogenicity with slightly impaired visualization of the diaphragm and the portal vein wall; and Grade 3, there is a severe increase of liver echogenicity with poor or no visualization of the diaphragm, the portal vein wall, and posterior part of the right liver lobe [17]. Patients who had a history of chronic hepatitis B and chronic hepatitis C were classified as Grade 4 (chronic liver disease). Other abnormal liver findings were classified as ‘other’ as one of the exclusion criteria. Diagnosis of fatty liver using liver ultrasound shows high accuracy compared with histological examination and is known as a cost-effective, safe, and easy method [18].

### 2.7. Measures of NAFLD: NAFLD Scoring Systems

#### 2.7.1. Fatty Liver Index (FLI)

The FLI was calculated as reported by Bedongni and colleagues [19] using the following formula:

FLI = [e^0.953×ln (triglycerides)+0.139×BMI + 0.718×ln (GGT)+0.053×waistcircumference−15.745^]/[1 + e^0.953×ln (triglycerides)+0.139×BMI+0.718×ln (GGT)+0.053×waistcircumference−15.745^] × 100

FLI ranges from 0 to 100, with an FLI < 30 ruling out fatty liver disease (sensitivity 87%) and FLI ≥ 60 suggesting the presence of fatty liver disease (specificity 86%) with a good diagnostic accuracy of 0.84 (95% CI: 0.81–0.87) [20]. In this study, groups were classified based on whether their score was <30 or ≥60.

#### 2.7.2. Hepatic Steatosis Index (HSI)

The HSI was calculated based on the report by Lee and colleagues [21]:

HSI = 8 × ALT/AST ratio + BMI (+2, if diabetes mellitus is present; +2, if female)

HSI values < 30 rule out fatty liver disease (sensitivity 93.1%) and HSI ≥ 36 suggests the presence of fatty liver disease (specificity 93.1%) with a good diagnostic accuracy of 0.93 (95% CI: 0.92–0.94) [21]. In this study, groups were classified based on whether their score was <30 or ≥36.

#### 2.7.3. Simple NAFLD Score (SNS)

The SNS was calculated using the formula as reported by Lee and colleagues [22]; details are provided in the Supplementary Table S1. For the SNS, ≥8 was selected as the cut-off point to define individuals with a high risk of NAFLD. In this study, groups were classified based on whether their score was ≥8.

#### 2.7.4. Comprehensive NAFLD Score (CNS)

The CNS was calculated using the formula reported by Lee and colleagues [22]; details are provided in the Supplementary Table S2. For the CNS, ≥40 was selected as the cut-off point to define individuals with a high risk of NAFLD. In this study, groups were classified based on whether their score was ≥40.

### 2.8. Ten-Year CVD Risk Calculation: Framingham Risk Score for Hard Coronary Heart Disease Individual Estimation of Participants’ 10-Year CVD Risk Was Performed Using the Framingham Risk Scores (FRS) Calculation Based on the National Cholesterol Education Program Guidelines

FRS is a sex-specific CVD prediction tool that estimates the 10-year risk of CVD and was developed based on data from the Framingham Heart Study [23]. Use of the FRS was recommended by the Adult Treatment Panel III guidelines and the score includes the following risk factors: age, sex, total cholesterol, HDL cholesterol, blood pressure, smoking, diabetes mellitus, the use of antihypertensive medication, and history of vascular disease (coronary artery disease, peripheral vascular disease, or stroke) [14]. Patients were assigned to three CVD risk groups according to revised National Cholesterol Education Program guidelines: low (10-year risk < 10%), moderate (10-year risk 10–20%), and high (10-year risk > 20%).

### 2.9. Statistical Analysis

All statistical analyses were performed according to the three CVD risk groups classified by the FRS. Categorical variables are presented as numbers with percentage, and continuous variables are presented as the mean ± standard deviation. Pearson’s chi-squared tests and ANOVAs were used for comparing groups. To determine the relationship between NAFLD diagnostic tools and CVD risk groups, multinomial logistic regression analysis was performed and adjusted for covariates. Each odds ratio (OR) was reported together with its 95% CI. In addition, receiver operating characteristic (ROC) curve analysis was performed to confirm the sensitivity and specificity of CVD risk prediction according to each NAFLD diagnostic tool and the AUC for each tool was calculated. ROC curve analysis was performed using MedCalc software version 19.2.1, and IBM SPSS statistics 25.0 was used for all other analysis. A *p*-value of <0.05 was defined as statistically significant.

## 3. Results

### 3.1. Baseline Demographic, Clinical, and Biochemical Characteristics of the Study Subjects According to CVD Risk Groups

Of 28,897 patients, 5521 were excluded, meaning 23,376 patients (13,583 males and 9793 females; mean age, 50.19 years) were included in the analysis. Participants were classified according to CVD risk groups; baseline demographic, clinical, and biochemical characteristics of the study subjects are presented in Table 1. Using the FRS, 15,760 (67.4%) subjects were categorized as low risk of CVD (<10% risk), 5019 (21.5%) were categorized as intermediate risk (10–20% risk), and 2597 (11.1%) were categorized as high risk (>20% risk). A total of 13,583 (58.1%) subjects were male and the proportion of males increased with increasing cardiovascular risk. The mean age and components of the obesity metabolic index (e.g., BMI, waist circumference, and the percentage of participants who smoked and drank alcohol) were higher in the high-risk group. The proportion of subjects who performed regular exercise was not significantly different between risk groups. With higher cardiovascular risk, systolic blood pressure, diastolic blood pressure, cholesterol, and fasting glucose levels increased. The prevalence of certain comorbidities increased significantly with higher cardiovascular risk, including hypertension, diabetes mellitus, and dyslipidemia.

### 3.2. Associations between Ultrasonographic Grading of Steatosis and NAFLD Scoring System Scores According to CVD Risk Groups

When fatty liver was classified using abdominal ultrasonography, the proportion of participants with mild and moderate-to-severe fatty liver disease in the low-risk group was 30.8% and 6.7%, respectively; in the intermediate-risk group, 50.9% and 14.9%, respectively; and in the high-risk group, 55.3% and 21.0%, respectively. As cardiovascular risk increased, there was a statistically significant increase in NAFLD severity (*p* < 0.001). The FLI showed similar results: the proportion of FLI scores between 30 and 60 and ≥60 in the low-risk group for CVD were 18.7% and 4.7%, respectively; in the intermediate-risk group, 37.9% and 10.2%, respectively; and in the high-risk group, 42.6% and 15.4%, respectively. Similar results were found for other scoring systems: the proportion of HSI scores between 30 and 36 and ≥36 in the low-risk group were 45.4% and 16.3%, respectively; in the intermediate-risk group, 51.1% and 24.8%, respectively; and in the high-risk group, 50.0% and 29.7%, respectively. The proportion of participants with a SNS ≥ 8 in low, intermediate, and high-risk groups was 27.8%, 53.2%, and 66.7%, respectively. The proportion of participants with a CNS ≥ 40 in the low, intermediate, and high-risk groups for CVD was 33.8%, 65.3%, and 75.9%, respectively. Among 23,376 patents, 2343 (10%) had moderate-to-severe fatty liver when assessed by abdominal ultrasonography. Of this group, 1051 (6.7%) patients were in the low-risk group, 747 (14.9%) were in the intermediate-risk group, and 545 (21%) were in the high-risk group. In addition, the score of each NAFLD scoring system significantly increased with increasing cardiovascular risk (*p* < 0.001). The FLI score increased from 20.69 to 37.09, the HSI score from 31.80 to 33.83, the SNS from 5.94 to 8.61, and the CNS from 32.19 to 63.45. Using the FLI score ≥ 60 criteria, NAFLD was identified in 1656 (7.1%) of the 23,376 patients; using the HSI score ≥ 36 criteria, NAFLD was identified in 4581 (19.6%) patients; using the SNS ≥ 8 criteria, NAFLD was identified in 8781 (37.6%) patients; and using the CNS ≥ 40 criteria, NAFLD was identified in 10,570 (45.2%) patients. With all four diagnostic tools, the number of patients fulfilling NAFLD diagnostic criteria was higher as cardiovascular risk increased (Table 2).

### 3.3. Relationship between Ultrasonography, NAFLD Diagnostic Tools, and Cardiovascular Risk Groups

Table 3 shows the relationship between ultrasonography, NAFLD diagnostic tools, and cardiovascular risk groups. Multinomial logistic regression analysis was conducted to determine the association between each diagnostic tool and cardiovascular risk. The ORs for ultrasonography, FLI, HSI, SNS, and CNS according to cardiovascular risk group are presented. After adjusting for covariates, the moderate-to-severe fatty liver group had an OR for intermediate cardiovascular risk of 2.73 (95% CI 2.27–3.28) and for high cardiovascular risk of 6.94 (95% CI 5.20–9.26). In the unadjusted analysis, the odds of a participant from the CNS ≥ 40 group having an intermediate cardiovascular risk were 3.69 times higher than a participant with a CNS < 40 (95% CI 3.45–3.95); the odds of high cardiovascular risk were 6.12 times higher (95% CI 5.62–6.81). In the adjusted analysis, a participant with an FLI score ≥ 60 was 2.59 times more likely to have an intermediate cardiovascular risk than a participant with an FLI score < 30 (95% CI 2.12–3.16); the odds of high cardiovascular risk were 6.36 times higher (95% CI 4.78–8.46).

### 3.4. The Prediction of High-Risk Cardiovascular Group According to NAFLD Diagnostic Tools

**Figure 1:**
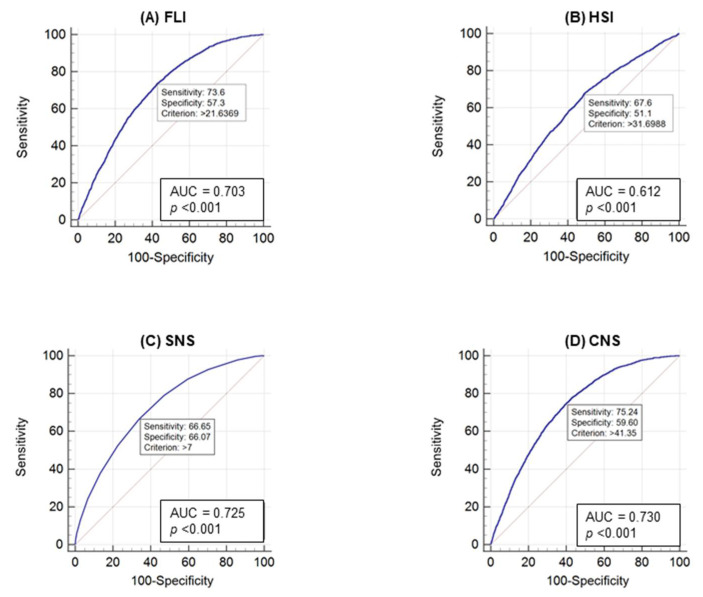
Receiver operating characteristic curve for the prediction of high-risk cardiovascular group inclusion according to NAFLD diagnostic tools. (**A**) Fatty Liver Index. (**B**) Hepatic Steatosis Index. (**C**) Simple NAFLD Score. (**D**) Comprehensive NAFLD Score. AUC, area under the curve.

Figure 1 illustrates the ROC curve analysis for the prediction of high-risk CVD group according to NAFLD diagnostic tools. All four diagnostic tools resulted in statistically significant ROC curves, of which the CNS had the highest AUC (0.730). The sensitivity and specificity were 75.2% and 59.6%, respectively, based on > 41.35 criterion. The SNS had the second highest AUC (0.725) with a sensitivity and specificity of 66.6% and 55.0%, respectively, based on >7 criterion. The AUC for the FLI was 0.703 (sensitivity, 73.6%, and specificity, 57.3%, based on >21.63 criterion) and the AUC for the HSI was 0.612 (sensitivity, 67.6%, and specificity, 51.1%, based on >31.69 criterion).

## 4. Discussion

We evaluated the association between ultrasonography, NAFLD scoring systems, and cardiovascular risk in a large Korean population (N = 23,376) using various fatty liver diagnostic tools (liver ultrasonography and four NAFLD scoring systems). We demonstrated that all tools were useful indicators of CVD risk and that fatty liver is an important independent risk factor for CVD; among the tools, ultrasonography, the FLI, and the CNS were found to be the most useful. We confirmed that both the severity of steatosis diagnosed by ultrasonography and the presence of fatty liver predicted through NAFLD scoring systems were associated with increased risk of CVD.

NAFLD is not only prevalent in Western societies but is an emerging problem in many Asian countries [24]. In Western countries, approximately 20–30% of adults in the general population have NAFLD and its prevalence increases substantially to 70–90% in people with metabolic diseases [25]. A large, population-based surveys in the Asia-Pacific region reported that the prevalence of NAFLD varied from 10–29% in population subgroups, depending on age, ethnicity, and gender [26]. The results of this study also revealed that the prevalence of NAFLD varied depending on the diagnostic method used: ultrasonography and the FLI, HSI, SNS, and CNS resulted in prevalences of 10.0%, 7.1%, 19.6%, 37.6%, and 45.2%, respectively, similar to previous studies. Considering the clinical significance and prevalence of NAFLD, having appropriate methods to aid the early identification of disease would have a significant impact on public health.

In this study, we assessed the relationship between ultrasonography, NAFLD diagnostic tools, and cardiovascular risk groups. For all tools, there was a correlation between score and cardiovascular risk even after adjusting for factors expected to influence the result, confirming NAFLD as an independent predictor of cardiovascular risk. Multinomial logistic regression, both before and after adjusting for covariates, showed that a higher degree of steatosis found via ultrasonography and a higher score from the FLI, HSI, SNS, and CNS were related to a higher risk of CVD. In both analyses, liver ultrasonography showed the strongest association. Among NAFLD diagnostic tools, ultrasonography, the FLI, and the CNS were most strongly associated with cardiovascular risk. Patients with moderate-to-severe fatty liver, diagnosed by ultrasonography, were 6.9 times more likely to be in the high-risk CVD group than patients with a normal liver ultrasound. If a patient’s FLI score was 60 or higher, the probability of being in the high-risk group for CVD was 6.3 times higher than a patient’s FLI score below 30; this association was significant after adjustment. However, the SNS and CNS showed lower OR values after adjustment in our analysis; this may be due to adjustment factors (e.g., age, gender) being adjusted both in the multinomial logistic regression analysis and in the calculation of cardiovascular risk. Nevertheless, significant associations were continually confirmed. These results suggest that liver ultrasound, the FLI, and the CNS have the strongest associations with CVD and that NAFLD is an independent risk factor for CVD. In agreement with this, Cuenza and colleagues have also recently shown that ultrasound-based grading of NAFLD severity is associated with an increased risk of CVD [27]. They suggest that ultrasound is a non-invasive, low risk, and easy-to-use procedure that can reliably detect the presence of NAFLD and help identify patients at risk of CVD.

Several previous studies have assessed the association between NAFLD and CVD risk and have demonstrated that NAFLD is an independent predictor of cardiovascular risk [28]. A previous study argued that the mechanisms by which NAFLD increases cardiovascular risk are very complex and involve multiple pathways, but that insulin resistance is the main determinant of NAFLD pathogenesis [10]. Another study reported evidence that NAFLD is strongly associated with increased CVD risk, and that NAFLD may not only be a marker, but also an early mediator, of atherosclerosis [29]. Aside from insulin resistance, NAFLD and atherosclerosis share unifying mechanisms involving pro-inflammatory, thrombogenic factors, and adipokines [30]. Systemic diffusion of cytokines and chemokines due to hepatic necro-inflammation triggers vascular damage and coagulation system abnormalities [10]. Expanded and inflamed visceral adipose tissue is the key mechanism linking NAFLD with increased cardiovascular risk. In steatohepatitis, pro-inflammatory and pro-atherogenic factors are released, which may lead to the development of insulin resistance and atherogenic dyslipidemia, pathologies critical in the development of both the metabolic syndrome and CVD [31,32].

The gold standard for diagnosing fatty liver disease is liver biopsy. However, due to its invasiveness, risk of complications, and high cost, it is not always feasible in the clinical setting. In this study, ROC analysis was used to confirm the usefulness of NAFLD in predicting CVD: we found that the FLI, HSI, SNS, and CNS were all useful. The CNS showed the highest AUC; the sensitivity and specificity for predicting high-risk CVD were 75.2% and 59.6%, respectively, based on >41.35 criterion. One drawback of ultrasound-based diagnosis of NAFLD is that it is highly operator-dependent, with significant inter- and intra-observer variability. In addition, ultrasonography is more expensive and takes more time than scoring systems [33], which use clinical and biochemical methods and have been shown to detect fatty liver disease with considerable accuracy in comparison with ultrasonography [19,21].

This study has several limitations. First, we defined excessive alcohol consumption based on grams of drinks per week (more than 210 g/week for males and 140 g/week for females). These data are an approximation, since we could not access study participants’ complete data on the amount of alcohol consumed. Second, our study population included only healthy Koreans who were actively participating in the medical checkup program, which could lead to selection bias. Third, the study is based on patients from a single local tertiary center, so findings may not be a good representation of the general population. Fourth, the study was cross-sectional, meaning the data are retrospective and descriptive in nature, and cannot confirm causality. Finally, the FRS that was used in this study overestimates cardiovascular risk in cohorts with low background risk of CVD in the Asian population [34]. Further prospective studies are necessitated to overcome this limitation.

Nevertheless, strengths of the current study merit consideration. First, to the best of our knowledge, our study is the first to report on the association of multiple, validated NAFLD scoring systems with the risk of CVD in a large-scale population. Second, this study was designed to include individuals free of clinically-evident prior vascular disease or other chronic disorders at baseline to minimize the possibility of reverse-causation bias.

## 5. Conclusions

In conclusion, our results demonstrate that higher scores on NAFLD scoring systems are associated with a higher risk of CVD; fatty liver is therefore an important, independent risk factor for CVD. Liver ultrasonography and the FLI and CNS scoring systems showed the strongest associations with cardiovascular risk. These widely-available NAFLD scoring systems could be a useful screening tool to identify patients who may benefit from lifestyle modification and appropriate CVD interventions in the long term. We believe that careful and routine screening for NAFLD and, simultaneously, prediction of cardiovascular risk, may lead to the early diagnosis and management of disease. By focusing on lifestyle modification and reducing risk factors, patients may demonstrate slower or no progression of liver disease and better cardiovascular outcomes. Further research is needed to determine whether NAFLD scoring systems could be a predictor of CVD in a clinical setting.

## Figures and Tables

**Table 1:** Baseline demographic, clinical, and biochemical characteristics of the study subjects according to CVD risk groups.

		Low-Risk Group (*n* = 15,760)	Intermediate-Risk Group (*n* = 5019)	High-Risk Group (*n* = 2597)	*p*-Value *
Age (years)		46.65 ± 9.01 ^a,b^	55.68 ± 7.80 ^a,c^	61.09 ± 7.29 ^b,c^	<0.001
Male gender		7161 (45.4)	4134 (82.4)	2288 (88.1)	<0.001
Hypertension		1589 (10.1)	1597 (31.8)	1386 (52.7)	<0.001
Diabetes mellitus		373 (2.4)	629 (12.5)	795 (30.6)	<0.001
Dyslipidemia		3348 (21.2)	1933 (38.5)	1273 (49.0)	<0.001
Body mass index (kg/m^2^)		23.33 ± 2.96 ^a,b^	24.63 ± 2.75 ^a,c^	24.92 ± 2.71 ^b,c^	<0.001
Height (cm)		164.86 ± 8.9 ^a,b^	167.38 ± 7.89 ^a^	166.92 ± 7.36 ^b^	<0.001
Weight (kg)		63.72 ± 11.47 ^a,b^	69.20 ± 10.46 ^a^	69.62 ± 10.01 ^b^	<0.001
Waist circumference (cm)		78.56 ± 9.10 ^a,b^	84.65 ± 7.79 ^a,c^	86.42 ± 7.75 ^b,c^	<0.001
Systolic blood pressure (mmHg)		117.38 ± 13.16 ^a,b^	128.33 ± 14.80 ^a,c^	136.92 ± 16.73 ^b,c^	<0.001
Diastolic blood pressure (mmHg)		71.42 ± 10.17 ^a,b^	78.74 ± 10.73 ^a,c^	82.22 ± 11.58 ^b,c^	<0.001
Smoking	Current	2247 (14.3)	1992 (39.7)	1579 (60.8)	<0.001
	Former	1249 (7.9)	531 (10.6)	207 (8.0)	<0.001
	Never	12,264 (77.8)	2496 (49.7)	811 (31.2)	<0.001
Moderate alcohol drinker		8327 (52.8)	3004 (59.9)	1558 (60.0)	<0.001
Regular exercise		3942 (25.0)	1253 (25.0)	610 (23.5)	0.242
Menopause		4319 (50.2)	533 (60.2)	175 (56.6)	<0.001
WBC (×10^3^/uL)		5.518 (1.58) ^a,b^	6.002 (1.659) ^a,c^	6.320 (1.766) ^b,c^	<0.001
hsCRP (mg/dL)		0.11 (0.35) ^a,b^	0.14 (0.38) ^a,c^	0.19 (1.07) ^b,c^	<0.001
AST (IU/L)		22.45 (11.21) ^a,b^	25.03 (11.67) ^a^	24.68 (9.48) ^b^	<0.001
ALT (IU/L)		21.47 (17.68) ^a,b^	25.93 (19.48) ^a^	25.50 (16.71) ^b^	<0.001
GGT (IU/L)		27.46 (31.36) ^a,b^	38.81 (44.97) ^a^	37.81 (33.67) ^b^	<0.001
ALP (IU/L)		61.75 (17.29) ^a,b^	66.57 (17.70) ^a,c^	69.75 (42.48) ^b,c^	<0.001
Total bilirubin (mg/dL)		0.79 (0.38) ^a^	0.84 (0.36) ^a,c^	0.80 (0.34) ^c^	<0.001
Creatinine (mg/dL)		0.81 (0.22) ^a,b^	0.90 (0.18) ^a,c^	0.93 (0.2) ^b,c^	<0.001
Total cholesterol (mg/dL)		192.79 (34.46) ^a,b^	200.23 (36.97) ^a,c^	202.87 (38.85) ^b,c^	<0.001
Triglycerides (mg/dL)		114.12 (70.52) ^a,b^	150.29 (96.70) ^a,c^	170.01 (113.53) ^b,c^	<0.001
HDL cholesterol (mg/dL)		59.26 (14.91) ^a,b^	51.44 (12.88) ^a,c^	47.95 (12.07) ^b,c^	<0.001
LDL cholesterol (mg/dL)		119.81 (31.27) ^a,b^	129.51 (33.43) ^a,c^	132.05 (34.87) ^b,c^	<0.001
Fasting plasma glucose (mg/L)		94.11 (15.04) ^a,b^	103.49 (24.61) ^a,c^	112.92 (35.36) ^b,c^	<0.001
Uric acid (mg/dL)		4.95 (1.38) ^a,b^	5.57 (1.34) ^a^	5.61 (1.41) ^b^	<0.001

Data are expressed as the mean ± standard deviation or number (%). * ANOVA for continuous variables and Pearson’s chi-squared test for discrete variables. ^a–c^ Post-hoc analysis using Scheffe’s method. Abbreviations: WBC, white blood cell; hsCRP, high sensitivity C-reactive protein; AST, aspartate aminotransferase; ALT, alanine aminotransferase; GGT, gamma-glutamyl transferase; ALP, alkaline phosphatase; HDL, high-density lipoprotein; LDL, low-density lipoprotein.

**Table 2:** Association between ultrasonographic grading of steatosis and the Fatty Liver Index (FLI), Hepatic Steatosis Index (HSI), Simple NAFLD Score (SNS), and Comprehensive NAFLD Score (CNS) according to cardiovascular risk groups.

	Low-Risk Group (*n* = 15,760)	Intermediate-Risk Group (*n* = 5019)	High-Risk Group (*n* = 2597)	*p*-Value *
USG				
Normal Mild Moderate-to-severe	9854 (62.5) 4855 (30.8) 1051 (6.7)	1717 (34.2) 2555 (50.9) 747 (14.9)	617 (23.8) 1435 (55.3) 545 (21.0)	<0.001
FLI	20.69 ± 18.05	32.38 ± 19.58	37.09 ± 20.63	<0.001
FLI < 30 30 ≤ FLI < 60 FLI ≥ 60	12,069 (76.6) 2944 (18.7) 747 (4.7)	2607 (51.9) 1902 (37.9) 510 (10.2)	1091 (42.0) 1107 (42.6) 399 (15.4)	<0.001
HSI	31.80 ± 4.63	33.26 ± 4.64	33.83 ± 4.79	<0.001
HSI < 30 30 ≤ HSI < 36 HSI ≥ 36	6032 (38.3) 7161 (45.4) 267 (16.3)	1210 (24.1) 2566 (51.1) 1243 (24.8)	528 (20.3) 1298 (50.0) 771 (29.7)	<0.001
SNS	5.94 ± 2.55	7.72 ± 2.44	8.61 ± 2.58	<0.001
SNS < 8 SNS ≥ 8	11,381 (72.2) 4379 (27.8)	2348 (46.8) 2671 (53.2)	866 (33.3) 1731 (66.7)	<0.001
CNS	32.19 ± 29.96	54.68 ± 29.44	63.45 ± 28.40	<0.001
CNS < 40 CNS ≥ 40	10,440 (66.2) 5320 (33.8)	1741 (34.7) 3278 (65.3)	625 (24.1) 1972 (75.9)	<0.001

Data are expressed as the mean ± standard deviation or number (%). * ANOVA for continuous variables and Pearson’s chi-squared test for discrete variables. Abbreviations: NAFLD, nonalcoholic fatty liver disease; USG, ultrasonography; FLI, Fatty Liver Index; HSI, Hepatic Steatosis Index; SNS, Simple NAFLD Score; CNS, Comprehensive NAFLD Score.

**Table 3:** Relationship between ultrasonography, NAFLD diagnostic tools, and cardiovascular risk groups.

NAFLD Diagnostic Tool	Cardiovascular Risk Group			
	OR (95% CI)			
	Intermediate	High	Intermediate	High
	Unadjusted		Adjusted *	
USG				
Normal	1.00	1.00	1.00	1.00
Mild	1.83 (1.62–2.05)	4.72 (4.27–5.21)	1.83 (1.62–2.05)	2.99 (2.45–3.64)
Moderate-to-severe	2.73 (2.27–3.28)	8.28 (7.26–9.44)	2.73 (2.27–3.28)	6.94 (5.20–9.26)
p-for trend	<0.001	<0.001	<0.001	<0.001
FLI				
FLI < 30	1.00	1.00	1.00	1.00
30≤ FLI < 60	2.99 (2.78–3.21)	4.16 (3.79–4.56)	2.04 (1.80–2.30)	3.42 (2.85–4.10)
FLI ≥ 60	3.16 (2.80–3.56)	5.90 (5.15–6.77)	2.59 (2.12–3.16)	6.36 (4.78–8.46)
p-for trend	<0.001	<0.001	<0.001	<0.001
HIS				
HSI < 30	1.00	1.00	1.00	1.00
30≤ HSI < 36	1.78 (1.65–1.92)	2.07 (1.86–2.30)	1.38 (1.23–1.55)	1.68 (1.41–2.02)
HSI > 36	2.41 (2.20–2.64)	3.43 (3.04–3.86)	1.68 (1.43–1.98)	2.43 (1.90–3.12)
p-for trend	<0.001	<0.001	<0.001	<0.001
SNS				
SNS < 8	1.00	1.00	1.00	1.00
SNS ≥ 8	2.95 (2.76–3.15)	5.19 (4.75–5.67)	1.15 (1.00–1.31)	1.29 (1.06–1.58)
p-for trend	<0.001	<0.001	<0.001	<0.001
CNS				
CNS < 40	1.00	1.00	1.00	1.00
CNS ≥ 40	3.69 (3.45–3.95)	6.19 (5.62–6.81)	1.89 (1.69–2.11)	3.05 (2.56–3.62)
p-for trend	<0.001	<0.001	<0.001	<0.001

* Adjusted for age, gender, hypertension treatment, diabetes mellitus treatment, dyslipidemia treatment, alcohol, smoking, and obesity (BMI ≥ 25 kg/m^2^). Abbreviations: OR, odds ratio; NAFLD, nonalcoholic fatty liver disease; FLI, Fatty Liver Index; CI, confidence interval; HSI, Hepatic Steatosis Index; SNS, Simple NAFLD Score; CNS, Comprehensive NAFLD Score.

## Data Availability

No new data were created or analyzed in this study. Data sharing is not applicable to this article.

## Supplementary Materials

The following are available online at https://www.mdpi.com/article/10.3390/healthcare9070899/s1, Table S1: Simple NAFLD score, Table S2: Comprehensive NAFLD score.

## Abbreviations

ALT, alanine transaminase; AST, aspartate transaminase; BMI, body mass index; CNS, comprehensive NAFLD score; CVD, cardiovascular disease; FLI, fatty liver index; FRS, Framingham risk scores; HDL, high-density lipoprotein; HSI, hepatic steatosis index; LDL, low-density lipoprotein; NAFLD, nonalcoholic fatty liver disease; ROC, receiver operating characteristic; SNS, simple NAFLD score.
