# Supplementary material for: Prediction of Cardiovascular Risk Using Nonalcoholic Fatty Liver Disease Scoring Systems
Source: Healthcare (Basel). 2021 Jul 15;9(7):899. doi: 10.3390/healthcare9070899 (PMC8303122; doi:10.3390/healthcare9070899)
Supplement: Supplementary file 1 [file healthcare-09-00899-s001.zip › healthcare-1278873-supplementary.pdf]

Simple NAFLD score (SNS) and comprehensive NAFLD score (CNS)

Supplementary Table S1. Simple NAFLD score.

<table>
<tr><th rowspan="2">Questions</th><th colspan="2">Score</th></tr>
<tr><th>Male</th><th>Female</th></tr>
<tr><td>Age (yrs)</td><td colspan="2"></td></tr>
<tr><td><35</td><td colspan="2">0</td></tr>
<tr><td>≥35</td><td colspan="2">2</td></tr>
<tr><td>Waist circumference (cm)</td><td></td><td></td></tr>
<tr><td><80 (M), < 75 (F)</td><td>0</td><td>0</td></tr>
<tr><td>80-89.9 (M), 75-84.9 (F)</td><td>2</td><td>1</td></tr>
<tr><td>90-99.9 (M), 85-94.9 (F)</td><td>3</td><td>2</td></tr>
<tr><td>≥ 100 (M), ≥ 95 (F)</td><td>4</td><td>3</td></tr>
<tr><td>Body mass index (kg/m^2)</td><td colspan="2"></td></tr>
<tr><td><23</td><td colspan="2">0</td></tr>
<tr><td>23-24.9</td><td colspan="2">1</td></tr>
<tr><td>25-26.9</td><td colspan="2">2</td></tr>
<tr><td>≥27</td><td colspan="2">3</td></tr>
<tr><td>Diabetes</td><td colspan="2">2</td></tr>
<tr><td>Dyslipidemia</td><td colspan="2">2</td></tr>
<tr><td>No regular exercise (physically inactive)</td><td colspan="2">1</td></tr>
<tr><td>Alcohol consumption(Men)</td><td>1</td><td>0</td></tr>
<tr><td>Menopause(Women)</td><td>0</td><td>1</td></tr>
<tr><td>Total score</td><td colspan="2"></td></tr>
</table>

Supplementary Table S2. Comprehensive NAFLD score.

NAFLD = 1 / (1 + exp(−x)) × 100
if male,<br>x = 0.016 × age + 0.182 × BMI + 0.089 × WC + 0.391× alcohol + 0.124 × exercise + 0.018 × fasting glucose + 0.773 × $\log_e$ (triglycerides) − 0.014 × HDL cholesterol + 0.145 × uric acid − 0.674 × $\log_e$ (AST) + 1.632 × $\log_e$ (ALT) − 21.695.
if female,<br>x = 0.320 × BMI + 0.044 × WC + 0.533 × diabetes + 0.016 × fasting glucose + 0.951 × $\log_e$ (triglycerides) − 0.015 × HDL cholesterol + 0.199 × uric acid − 0.645 × $\log_e$ (AST) + 1.302 × $\log_e$ (ALT) + 0.255 × menopause − 19.741.
